# Chromosome-level genome assembly of the Stoliczka’s Asian trident bat (*Aselliscus stoliczkanus*)

**DOI:** 10.1038/s41597-023-02838-0

**Published:** 2023-12-15

**Authors:** Linjing Lan, Xin Zhang, Shanxiu Yang, Lingjie Li, Xiuguang Mao

**Affiliations:** 1 School of Ecological and Environmental Sciences, East China Normal University, Shanghai, 200062 China; 2 Department of Histoembryology, Genetics and Developmental Biology, Shanghai Key Laboratory of Reproductive Medicine, Key Laboratory of Cell Differentiation and Apoptosis of Chinese Ministry of Education, Shanghai Jiao Tong University School of Medicine, Shanghai, 200025 China

**Keywords:** Evolutionary genetics, Genome

## Abstract

Stoliczka’s Asian trident bat (*Aselliscus stoliczkanus*) is a small-bodied species and very sensitive to climate change. Here, we presented a chromosome-level genome assembly of *A. stoliczkanus* by combining Illumina sequencing, Nanopore sequencing and high-throughput chromatin conformation capture (Hi-C) sequencing technology. The genome assembly was 2.18 Gb in size with 98.26% of the genome sequences anchored onto 14 autosomes and two sex chromosomes (X and Y). The quality of the genome assembly is very high with a contig and scaffold N50 of 72.98 and 162 Mb, respectively, Benchmarking Universal Single-Copy Orthologs (BUSCO) score of 96.6%, and the consensus quality value (QV) of 47.44. A total of 20,567 genes were predicted and 98.8% of these genes were functionally annotated. Syntenic blocks between *A. stoliczkanus* and *Homo sapiens*, together with previous comparative cytogenetic studies, provide valuable foundations for further comparative genomic and cytogenetic studies in mammals. The reference-quality genome of *A. stoliczkanus* contributes an important resource for conservative genomics and landscape genomics in predicting adaptation and vulnerability to climate change.

## Background & Summary

Stoliczka’s Asian trident bat (*Aselliscus stoliczkanus*) is one of the three species in the genus *Aselliscus* (Hipposideridae)^1^ and widely distributes in Southeast Asia, including southeastern China, Myanmar, Thailand, Laos, Vietnam, and the Peninsular Malaysia^2–5^. It is assessed as least concern (LC) by the International Union for Conservation of Nature (IUCN)^2,5^. However, as a small-bodied species, *A. stoliczkanus* is very sensitive to climate change, in particular to humidity^6^. Thus, although *A. stoliczkanus* has a wide distribution and is listed as LC on the IUCN Red list, previous field surveys of cave bats revealed that the population size of this species has declined rapidly in recent years^7,8^. So, *A. stoliczkanus* has been recognized as near threatened (NT) on China Species Red List^9,10^. This species can be regarded as a valuable bio-indicator to climate change, as other bat species^11,12^. However, up to now, no reference-quality genome has been generated for *A. stoliczkanus*, which is valuable for the assessment of its conservation status and effective protection management^13,14^.

*A. stoliczkanus* with diploid chromosome number (2n) of 30 is one of the first bat species whose chromosomes were flow-sorted to be used to generate a whole set of chromosome-specific painting probes^15^. As far as we know, chromosome-specific paints of *A. stoliczkanus* have been used in comparative cytogenetic studies on four bat families, including Hipposideridae^16^, Rhinolophidae^15,17,18^, Megadermatidae^19^, and Vespertilionidae^20^. By integrating comparative chromosomal maps between *A. stoliczkanus* (Asto) and *Myotis myotis* (MMY)^21^, as well as between *A. stoliczkanus* and *Homo sapiens* (HSA)^15^, conserved syntenies of species from 10 bat families have been generated, which have been used to investigate the chromosomal evolution and predict the ancestral karyotype of Chiroptera^22,23^. Thus, a reference-quality genome of *A. stoliczkanus* and further whole-genome alignments between *A. stoliczkanus* and the other two species (MMY and HSA) will be very useful to confirm the genome syntenic blocks identified in previous comparative cytogenetic studies.

**Fig. 1:**
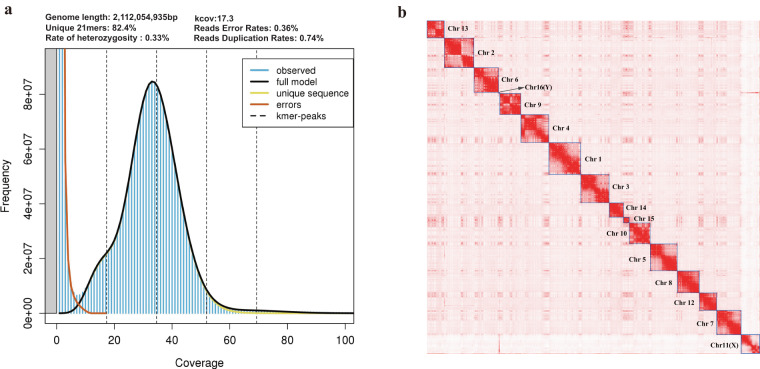
(**a**) Genomescope profile for 21-mers based on Illumina short-reads. (**b**) Hi-C contact map for the genome assembly.

**Fig. 2:**
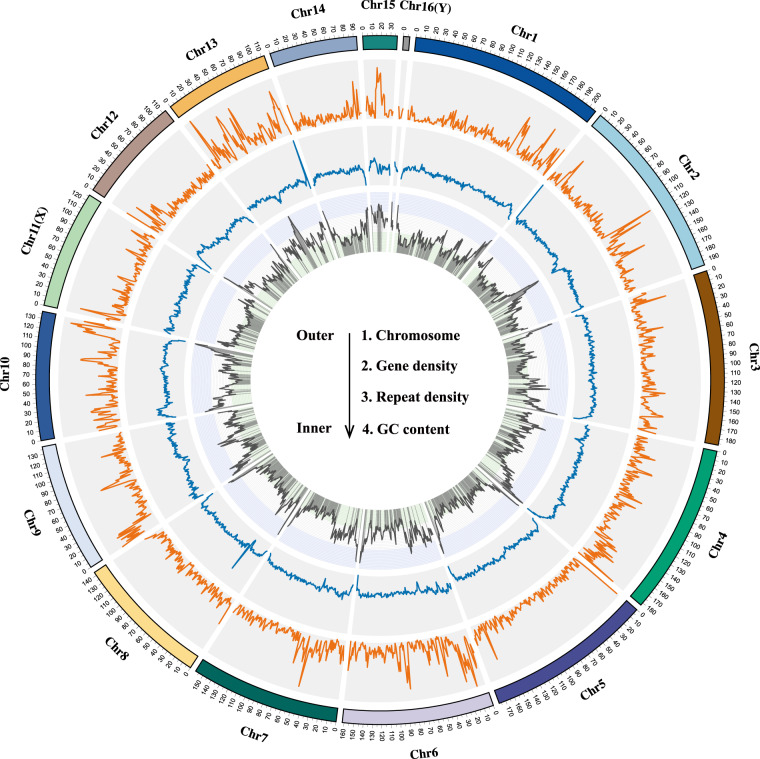
Circos showing the genomic structure of *A. stolizkanus* including chromosome, gene density, repeat density, and GC content (%) from the outer circle to inner one.

**Fig. 3:**
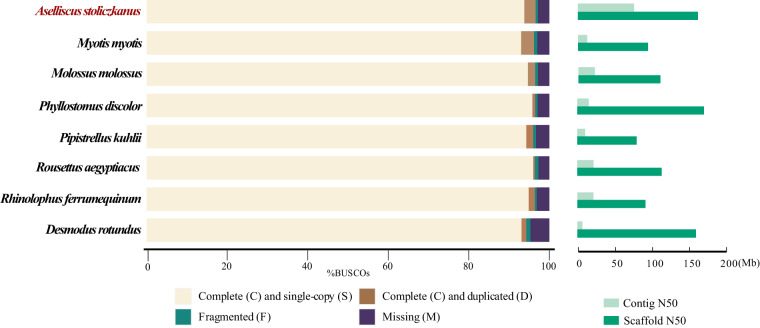
The contiguity of the genome assembly revealed by Benchmarking Universal Single-Copy Orthologs (BUSCO) score and N50 of contig and scaffold. Genome assembly of *Aselliscus stoliczkanus* was shown in red.

In this study, we presented a chromosome-level genome assembly for *A. stoliczkanus* using a combination of Illumina short-read sequencing (99.76 Gb), Nanopore long-read sequencing (203.07 Gb) and high-throughput chromatin conformation capture (Hi-C) sequencing (222.41 Gb) (Table 1). The final genome assembly size was 2.18 Gb with the contig and scaffold N50 of 72.98 Mb and 162 Mb, respectively (Table 2). Consistent with the karyotype reported in previous studies^15,21^, the final chromosome-level genome assembly includes 14 autosomes, X and Y chromosome (Figs. 1, 2 and Table 3), containing 99.66% of the total genome assembly. Our genome assembly of *A. stolizkanus* is comparable to other bat genomes (Fig. 3) and can be reliably used in further comparative genomics. In the genome assembly of *A. stoliczkanus* we detected 760.02 Mb (34.75% of the genome) repetitive elements (Table 4). After masking repetitive elements, a total of 20,320 protein-coding genes were predicted and 98.8% of them were functionally annotated (Table 5).

**Table 1:** Statistics of the genome sequencing data for *A. stoliczkanus*.

Library	Reads number	Raw data (Gb)	Clean data (Gb)	Reads N50 (bp)
Illumina	676,766,316	101.52	99.76	/
ONT	12,499,720	204.67	203.07	24,918
Hi-C	1,498,397,190	224.76	222.41	/

**Table 2:** Statistics of the genome assembly and genome evaluation for *A. stoliczkanus*.

Feature	Value
**Genome assembly statistics**	
Estimated genome size (bp)	2,112,045,935
The size of assembly (bp)	2,187,325,338
Contigs number	235
Contigs N50 (bp)	72,975,460
Scaffolds number	191
Scaffolds N50 (bp)	162,004,500
GC content (%)	41.1
Total base on chromosomes (bp)	2,179,935,021
Hi-C loading rates (%)	99.66
**Genome assembly evaluation**	
Nanopore reads mapping rates (%)	99.96
Illumina reads mapping rates (%)	99.68
Base pair accuracy (QV)	47.44
BUSCO score (%)	96.6

**Table 3:** The length of each chromosome in the genome assembly of *A. stoliczkanus*.

Chromosome ID	Chromosome ID in previous study^15^	Chromosome Length (bp)
Chr1	Chr1	207,901,853
Chr2	Chr3	194,675,023
Chr3	Chr4	186,534,500
Chr4	Chr2	183,023,020
Chr5	Chr7	177,415,154
Chr6	Chr5	162,004,500
Chr7	Chr6	158,905,207
Chr8	Chr8	143,714,873
Chr9	Chr9	137,972,201
Chr10	Chr11	137,439,702
Chr11(X)	X	126,483,028
Chr12	Chr12	113,898,000
Chr13	Chr10	113,190,041
Chr14	Chr13	93,423,647
Chr15	Chr14	36,716,714
Chr16(Y)	Y	6,637,558

**Table 4:** Summary of repetitive elements in the genome assembly of *A. stoliczkanus*.

Type of elements	Number of elements	Length (bp)	Percentage of genome (%)
SINEs	357,754	51,157,424	2.34
LINEs	829,135	432,550,674	19.78
LTR elements	428,008	129,647,761	5.93
DNA transposons	591,882	106,858,154	4.89
Rolling-circles	16,062	1,164,855	0.05
Unclassified	17,137	2,842,690	0.13
**Total interspersed repeats**	2,239,978	724,221,558	33.12
Satellites	8,556	1,279,207	0.06
Simple repeats	698,500	29,828,252	1.36
Low complexity	93,923	4,693,480	0.21
**Total tandem repeats**	800,979	35,800,939	1.63
**Total**	3,040,957	760,022,497	34.75

**Table 5:** Summary of gene structures and function in *A. stoliczkanus*.

Item	Number	Average length (bp)
Gene	20,567	45,892.69
Exon	422,449	279.57
CDS	403,817	166.47
**Database**	**Number**	**Percentage (%)**
Pfam	17,687	86
SwissProt	19,253	93.61
NR	18,068	87.85
eggNOG	19,776	96.15
All	20,320	98.8

**Fig. 4:**
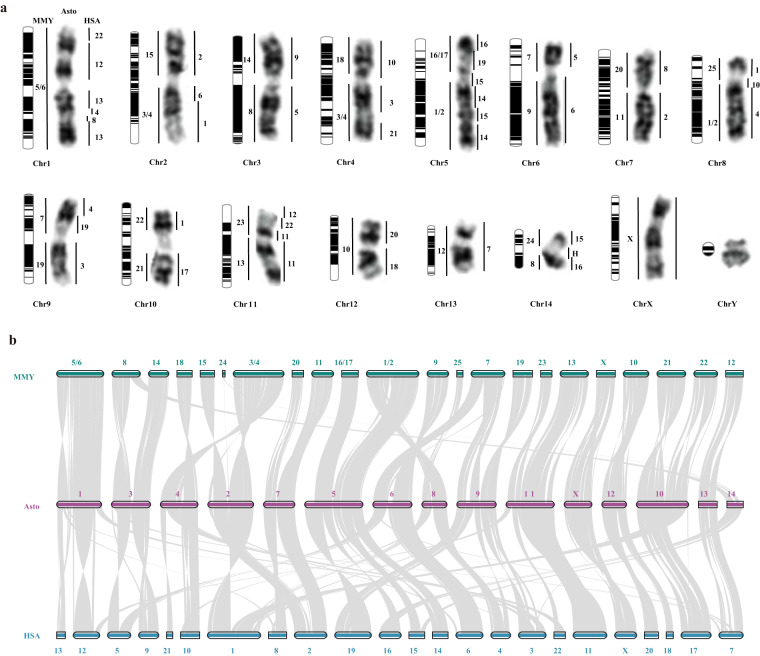
(**a**) G-banded karyotype of *A. stolizkanus* with syntenic blocks between *A. stolizkanus* (Asto) and two other species (*Myotis myotis*: MMY; *Homo sapiens*: HSA_)_ identified in previous comparative cytogenetic studies^15^_._ The capital letter “H” in chromosome 14 represents heterochromatin. The diagram for each chromosome is also shown on the left and the black color in the diagram represents the regions in which the GC content of a 1-Mb window is lower than the average GC content of the chromosome. (**b**) Genomic synteny and collinearity among Asto, MMY and HSA. Chromosomes in the genome assembly of MMY were numbered on the basis of previously published flow karyotype of this species^21^.

By performing genomic synteny analysis, we validated the results of previous comparative cytogenetic studies between *A. stoliczkanus* and the other two species^15,21^ (Fig. 4 and Table 3). This consistency also supports the high quality of *A. stoliczkanus* genome assembly and annotation generated in this study. Our current genomic resource of *A. stoliczkanus* will be very useful for the assessing of its conservation status and designing effective protection strategies in the future. In addition, syntenic blocks identified between *A. stoliczkanus* and other species will provide valuable foundations for further comparative genomic and cytogenetic studies in bats and also in mammals.

## Methods

### Sample collection and sequencing

An adult male *A. stolizkanus* was collected in November 2018 at Xiaogou cave in Yunnan province, China (25°03′16.7″N, 103°22′52.5″E). Bat was euthanized by cervical dislocation and four tissues (muscle, heart, brain, and liver) were sampled with RNase-free tubes. All tissues were frozen immediately in liquid nitrogen and then were stored in a −80 °C freezer. Sampling and tissue collection procedures were approved by the National Animal Research Authority, East China Normal University (approval ID bf20190301).

For genome sequencing, genomic DNA extracted from muscle with DNeasy kits (Qiagen) was used to construct three different sequencing libraries. First, Nanopore long read library (DNA fragment >20 kb) was constructed with the SQK-LSK109 kit (Oxford Nanopore Technologies, UK) and sequenced on a PromethION sequencer (Oxford Nanopore). The quality of Nanopore reads was assessed using Nanoplot v1.40.2^24^ and results have been shown in supplementary Table S1. Then reads were further trimmed by Nanofilt v2.8.0^24^ with default parameters. Second, for genome survey and error correction of Nanopore data, Illumina short read library (DNA fragment ~350 bp) was constructed with the NEBNext Ultra DNA library Pre-Kit and sequenced on the Illumina Novaseq. 6000 platform (pair-end 150 bp). Illumina short reads were assessed and trimmed by fastp v0.23.2^25^ (-q 20 -w 5 -u 40 -n 5). Third, to generate a chromosome-level genome, we created the Hi-C library with the Truseq Nano DNA library Kit and the restriction endonuclease DpnII following procedures described previously^26^ and sequenced it on the Illumina Hiseq platform (pair-end 150 bp). Hi-C reads were also processed by fastp v0.23.2^25^.

For transcriptome sequencing, the total RNA of each tissue (heart, brain and liver) was extracted using TRIzol (Life Technologies Corp., Carlsbad, CA, USA). A total of three RNA sequencing libraries were constructed using NEBNext® UltraTM RNA Library Prep Kit for Illumina® (NEB, USA) and sequenced on the Illumina HiSeq X Ten platform (paired-end 150 bp). RNA-seq reads were trimmed using TRIMMOMATIC v0.38^27^ with default parameters.

### Genome assembly

Jellyfish v2.2.10^28^ was used to construct the k-mer count histogram (k = 21) based on 99.76 Gb clean short read data. Then, the genome size, heterozygosity and percentage of repeat content were estimated using GenomeScope v2.0^29^. Genomic contig assembly was performed based on 203.07 Gb Nanopore long reads using NextDenovo^30^ software (https://github.com/nextomics/nextdenovo). Then Nextpolish v1.4.1^31^ was applied to polish the assembly with both Nanopore long reads and Illumina short reads. Redundant contigs were removed using Purge_Dups v1.2.5^32^ with default settings. A total of 222.41 Gb clean Hi-C reads were then mapped to the contig assembly using Juicer v1.6^33^ and chromosome construction was conducted using the 3D-DNA pipeline^34^ with default settings. We further used Juicebox Assembly Tools^35^ to manually correct the chromatin contact matrix and built the Hi-C interaction heatmap. The final genome assembly was generated by 3D-DNA post-review pipeline based on the corrected assembly file above.

To evaluate the quality of the genome assembly, we first assessed its integrity using Benchmarking Universal Single-Copy Orthologs (BUSCO v5.2.2)^36^ with a database of mammals (mammalia_odb10). Second, the Nanopore long reads and Illumina short reads were mapped to the genome assembly using minimap2 v2.24-r1122^37^ and bwa v0.7.17-r1188^38^ with default parameters, respectively. We then estimated the mapping rates using SAMtools v1.16.1^39^. Third, the accuracy of our genome assembly was assessed by calculating the consensus quality (QV) using MERQURY v1.3^40^ based on Illumina short reads and k-mers.

### Identification of sex chromosomes

We identified the X chromosome of *A. stolizkanus* by aligning its genome assembly against the genome of *R. ferrumequinum* (NCBI accession number: GCF_004115265.2) whose X chromosome had been identified^41^. Y chromosome of *A. stolizkanus* was identified by performing blastn searches with Y-linked genes in the mammals (*USP9Y* and *UTY*)^42^.

### Repeat annotation

We annotated the repeat sequences in the *A. stolizkanus* genome using both de novo and homology-based prediction methods. A de novo repeat library was first created using RepeatModeler (https://github.com/Dfam-consortium/RepeatModeler) (the ‘-LTRStruct’ option), which was merged with the bat repeat libraries^43^, Repbase (http://www.girinst.org/repbase) and Dram database to generate a final custom repeat library. RepeatMasker v4.1.2^44^ was then used to perform repeat sequence annotation with the custom repeat library. A total of 760.02 Mb (34.75% of the genome) repetitive elements were identified, of which 33.12% was transposable elements (TEs), including LINE (19.78%), LTR (5.93%), SINE (2.34%), DNA transposons (4.89%), Rolling-circles (0.13%) and unclassified TEs (0.13%) (Table 4).

### Gene annotation

We used three methods to predict protein-coding genes, including ab initio prediction, transcriptome-based prediction and homology-based prediction. The BRAKER2 v2.5.2 pipeline^45^ was applied to perform ab initio prediction using de novo, homology-based protein and RNA-Seq evidence. For transcriptome-based prediction, a total of 21.3 Gb RNA-seq reads from four tissues were mapped to the genome using HISAT2 v2.2.1^46^ with default parameters and the transcriptome was assembled using STRINGTIE v2.2.1^47^. The open reading frames (ORFs) were predicted by TransDecoder v5.5.0 (https://github.com/TransDecoder/TransDecoder/). Homology-based prediction was performed using GEMOMA v1.9.0^48,49^ based on protein sequences of 11 species including six bats (*Rousettus aegyptiacus*, *Rhinolophus ferrumequinum*, *Pipistrellus kuhlii*, *Phyllostomus discolor*, *Myotis myotis*, and *Molossus molossus*) and five other mammals (*Felis catus, Bos taurus*, *Sus scrofa*, *Mus musculus*, and *Homo sapiens*). Then, we used EVidenceModeler v2.0.0^50^ to combine genes predicted by the three methods with a weighted consensus (ABINITIO_PREDICTION AUGUSTUS 1, TRANSCRIPT Cufflinks 12, OTHER_PREDICTION GeMoMa 10, OTHER_PREDICTION transdecoder 12). Finally, two rounds of PASA v2.4.1^51^ were conducted to update the EVM result using the transcriptome de novo assembled by Trinity v2.13.2^52^ under the default settings. We performed functional annotation by searching sequences of protein-coding genes against the Uniprot database and nonredundant protein sequence database (NR) using DIAMOND (blastp -e 1e-5)^53^, and the eggNOG database using EggNOG-mapper^54^. In addition, INTERPROSCAN^55^ (-appl Pfam -iprlookup -goterms) was employed to obtain protein domains and motifs and gene ontology (GO).

### Genome synteny

Genomic synteny analyses were performed between *A. stoliczkanus* and two other species (*M. myotis* and *H. sapiens*). We first conducted the pairwise alignment of these chromosome-level genomes using LAST software v1410^56^, then we identified and visualized the synteny blocks using MCscan (python version) with default parameters. High collinearity was observed across the three species (Fig. 4b) and synteny blocks identified here could be used to validate the results of previous comparative cytogenetic studies.

## Data Records

All raw sequencing data that were used for genome assembly and annotation have been deposited into the National Center for Biotechnology Information (NCBI) with accession number SRR25459631^57^ and SRR25476260^58^ for Illumina sequencing data, SRR25470059^59^ and SRR25470058^60^ for Nanopore sequencing data, SRR25490035^61^ for Hi-C sequencing data, SRR25461847^62^, SRR25461853^63^, and SRR25461918^64^ for transcriptome Illumina sequencing data. The final genome assembly and gene annotation results have been deposited in Figshare^65^ and in the GenBank database of NCBI with accession number JAWWOG000000000^66^.

## Technical Validation

High quality of the genome assembly of *A. stoliczkanus* was supported by multiple evaluation methods (BUSCO score: 96.6%; QV value: 47.44; mapping rates of Illumina short reads and Nanopore long reads: 99.68% and 99.96%, Table 2). In addition, the final assembled chromosome-level genome (99.66% of the total genome) contained the same number of chromosomes with the karyotype reported previously. Finally, highly homologous genomic segments between *A. stoliczkanus* and two other species (*M. myotis* and *H. sapiens*) revealed by genomic synteny analysis also supported the high quality of the genome assembly and annotation of *A. stoliczkanus*.

### Supplementary information

Table S1

## Data Availability

All commands and pipelines used in the data processing were all executed according to the manuals and protocols of the corresponding bioinformatics software. If no detailed parameters were provided, default parameters were used. The version of the software has been specified in the Methods section. No custom programming or coding was used.
