# Supplementary material for: Chromosome-level genome assembly of the Stoliczka’s Asian trident bat (Aselliscus stoliczkanus)
Source: Sci Data. 2023 Dec 15;10:902. doi: 10.1038/s41597-023-02838-0 (PMC10724287; doi:10.1038/s41597-023-02838-0)
Supplement: Supplementary file 1 — Table S1 [file 41597_2023_2838_MOESM1_ESM.pdf]

Table S1 The QC outputs of Nanopore sequencing reads used to generate the assembly.

	General summary
Mean read length	16,373.80
Mean read quality	11.3
Median read length	13,319.00
Median read quality	11.4
Number of reads	12,499,720.00
Read length N50	24,918.00
STDEV read length	13,779.90
Total bases	204,668,108,606.00
Number, percentage and megabases of reads above quality cutoffs	
>Q5	12499720 (100.0%) 204668.1Mb
>Q7	12499720 (100.0%) 204668.1Mb
>Q10	9273254 (74.2%) 161722.2Mb
>Q12	4701506 (37.6%) 88164.0Mb
>Q15	80299 (0.6%) 1176.8Mb
